# Does the Value of the Faecal Immunochemical Test (FIT) Result Matter, and Can This Be Used as a Triage Criterion for Symptomatic FIT-Positive Patients?

**DOI:** 10.7759/cureus.94372

**Published:** 2025-10-11

**Authors:** Firuza Soxibova, Neil Muscat, Neeraj Thapa, Naomi MacKenzie

**Affiliations:** 1 General Surgery, Wrightington, Wigan and Leigh NHS Foundation Trust, Wigan, GBR; 2 General Surgery, Hillingdon Hospitals NHS Foundation Trust, London, GBR; 3 General Surgery, Jersey General Hospital, St. Helier, JEY; 4 Colorectal Surgery, Wrightington, Wigan and Leigh NHS Foundation Trust, Wigan, GBR

**Keywords:** benign bowel pathology, colorectal cancer, faecal immunochemical test, screening, suspected colorectal cancer pathway

## Abstract

Background

The Faecal Immunochemical Test (FIT) is a cornerstone in both colorectal cancer (CRC) screening programs and referral pathways for symptomatic patients. The test identifies haemoglobin in stool and, when present, is used as a referral indicator on the suspected CRC pathway. Since its introduction, the number of referrals with a positive FIT has risen, stretching diagnostic capabilities to the limit. This study examines whether the value of the result is significant and, if so, can be used as part of the triage process.

Methods

This retrospective cohort study analysed patients who underwent FIT as part of a CRC screening program between June 2020 and November 2021 in Wrightington, Wigan and Leigh NHS Foundation Trust. A total of 293 patients contributed clinical data, including FIT scores, presenting symptoms, and diagnostic outcomes. The patients were stratified according to ranges of FIT scores (<10, 11-50, 51-100, 101-150, 151-200, >400). Their correlating outcomes, including the presence of cancer, benign findings (e.g., polyps and diverticular disease), and inflammatory conditions (e.g., colitis), were recorded.

Results

Out of the 293 patients included in the study, 223 were FIT-positive and 70 FIT-negative, with 20 refusing investigations in the FIT-negative group and six in the FIT-positive group.

Among the investigated patients, 25 were diagnosed with CRC; all cases were in the FIT-positive group, and none were found in the FIT-negative group. The sensitivity of FIT for CRC detection was 100% (95% CI: 86.7-100%), and the negative predictive value (NPV) was 100% (95% CI: 94.8-100%), confirming FIT's strength as a rule-out test in this cohort. The positive predictive value (PPV) of a positive FIT result for CRC was 11.2% (95% CI: 7.4-15.9%).

Of the 25 cancers, 13 (52%) had a FIT value of >400, and five (20%) had a FIT value of 11-50. In this cohort, 76% of CRC were in the sigmoid or rectum, with 63% of these cases associated with FIT values >400 and 15% falling within the lower FIT range of 11-50.

For the remaining FIT-positive patients without cancer, 123/198 (62.1%) had a FIT value of 11-50. Of these, 63 (51.2%) had either a polyp, diverticular disease, or colitis. Of the remaining cancer-negative patients, 30/198 (15.2%) had a FIT value of >400, of which 22 (73%) had benign pathology. When symptoms were analysed, most patients had a change in bowel habit, with rectal bleeding being the most typical symptom in the cancer group.

Conclusions

FIT is used widely to aid referral for investigating symptoms suggestive of bowel cancer. As more patients are being referred with positive FIT, there is a need to try to rationalise investigations. The premise of this study was to determine whether a low FIT value corresponded with a low chance of finding a cancer in symptomatic patients, thus aiding the triage process, reducing the numbers having unnecessary investigations, and relieving the burden on endoscopy units.

In this particular study, none of the FIT-negative patients had bowel cancer, confirming FIT's strong rule-out performance. However, as 20% of cancers occurred among patients with low-positive FIT results (11-50 µg/g), clinicians should remain cautious in interpreting low values as entirely reassuring. While most cancers were seen with FIT >400, PPV remained low overall (~11%), and high FIT scores did not always indicate malignancy.

## Introduction

Colorectal cancer (CRC) stands as one of the main burdens to the public health sector as the third most common malignancy and the second most frequent cause of death due to cancer in humans worldwide. Timely detection of the disease remains crucial to the prognosis because it has been determined that more than 90% of the cases have better survival rates if CRC is detected early [1]. Consequently, various screening modalities have been developed for the reduction of mortality rates by identifying CRC and precancerous lesions before progression, including colonoscopy, sigmoidoscopy, and stool-based assessments, among others. Among them, the Faecal Immunochemical Test (FIT) has become a preferred method in most CRC screening programs across the world because it is cost-effective and non-invasive.

FIT works by using antibodies that are designed to specifically recognise and bind to human haemoglobin in stool samples, providing a quantitative score and therefore categorising patients into various classes of risk. High scores on FIT are strongly associated with CRC and advanced adenomas, while lower scores are often related to benign disease or no disease at all [2]. Such risk stratification guides clinical decision-making, with high-risk patients needing urgent colonoscopy while those scoring low are kept under surveillance. Despite these merits, FIT has some weaknesses, especially in the detection of proximal cancers and lesions that bleed intermittently or minimally. Such cancers are associated with highly unfavourable outcomes; this thus calls for more advanced diagnostic techniques [3].

Aside from FIT scores, symptoms such as rectal bleeding, weight loss, changes in bowel habits, and iron deficiency anaemia (IDA) are essential for the diagnosis of CRC. Their predictive values vary, with some closely associated with malignancy and others overlapping with benign conditions such as diverticular disease and inflammatory bowel disease. The combination of symptomatology and FIT scores is a key factor in better risk stratification, reassuring us about the current diagnostic practices and their ability to guide the appropriate utilisation of diagnostic resources.

Furthermore, the development of biomarkers and molecular diagnostics is opening new perspectives for overcoming FIT limitations. Innovative approaches such as stool DNA tests, blood-based assays, and microbiome profiling show promising potential, especially for proximal cancers. The integration of these methods with FIT could significantly enhance the overall effectiveness of CRC screening programs, offering hope for improved screening and diagnosis in the future.

This study will assess the diagnostic efficiency of FIT scores for various risk categories, investigate symptoms associated with cancer positivity, and examine how FIT thresholds influence clinical decision-making and resource utilisation. This study aims to contribute to the refinement of CRC screening protocols by analysing clinical and diagnostic data to improve patient outcomes.

## Materials and methods

A retrospective cohort of 293 patients who underwent FIT evaluation between the time frame of June 1, 2020, and November 30, 2021, at Wrightington, Wigan and Leigh (WWL) NHS Foundation Trust were used for this study. All patients had been referred for colorectal clinic review via their general practitioner following FIT evaluation, primarily through the two-week wait (2WW) referral pathway, with symptoms including changes in bowel habit (CIBH) due to constipation or diarrhoea, rectal bleeding, weight loss, abdominal mass, or IDA. Anonymised patient data were extracted from electronic health records and compiled into a structured database containing information on presenting symptoms, FIT score range, imaging modalities performed, and corresponding diagnostic findings, including benign and malignant results with their anatomical locations. Patient presentations were stratified by symptom profile and by FIT range (<10, 11-50, 51-100, 101-150, 151-200, 201-250, 251-300, 301-350, 351-400, >400 µg Hb/g) as well as by the investigation modalities utilised (computed tomography (CT), colonoscopy, CT colonography (CTC), flexible sigmoidoscopy, and digital rectal examination (DRE)). Both benign and malignant findings were recorded, including lesion location and histological results. Statistical analyses were performed to assess correlations between FIT values, symptom profiles, investigation modalities, and diagnostic outcomes.

Inclusion and exclusion criteria

The study included patients 18 years and older who underwent FIT for CRC screening following symptomatic presentation to the clinic. Inclusion criteria mandated documented FIT results with follow-up investigations within a defined time frame and, for those who refused further investigations, clear documentation of the said outcome. Patients in whom the medical records are incomplete or who have a previous diagnosis of CRC were excluded. Also, individuals who had undergone recent colorectal procedures or were not able to provide a FIT specimen for health reasons were excluded from the study, as well as those with an existing diagnosis of CRC previously. 

Primary outcome

The primary outcome measure was a correlation between the FIT score and the presence of CRC.

Secondary outcome

Secondary outcome measures included correlations between FIT score and location of cancer (proximal colon vs. distal colon), symptom prevalence, presence of certain benign pathologies (polyps, inflammatory conditions, etc.), and determining investigation outcomes.

Statistical methods

All analyses were conducted using established statistical workflows to assess diagnostic performance and associations between variables. Sensitivity, specificity, positive predictive value (PPV), and negative predictive value (NPV) were calculated for each FIT category, with 95% confidence intervals (CIs) determined using the Wilson score method, which provides accurate interval estimates for proportions, particularly in small or unevenly distributed samples. Group comparisons between categorical variables, such as FIT ranges and cancer detection or symptom prevalence, were performed using the chi-squared test or Fisher's exact test when expected cell counts were small (<5), ensuring the reliable evaluation of associations. Odds ratios (ORs) with 95% CIs were calculated to quantify the strength and direction of relationships between FIT categories and diagnostic outcomes. Statistical significance was set at p<0.05. These methods were chosen to robustly characterise diagnostic accuracy and assess associations while accounting for sample size limitations and the categorical nature of the data.

## Results

Correlation between FIT score and accurate cancer diagnosis

**Figure 1:**
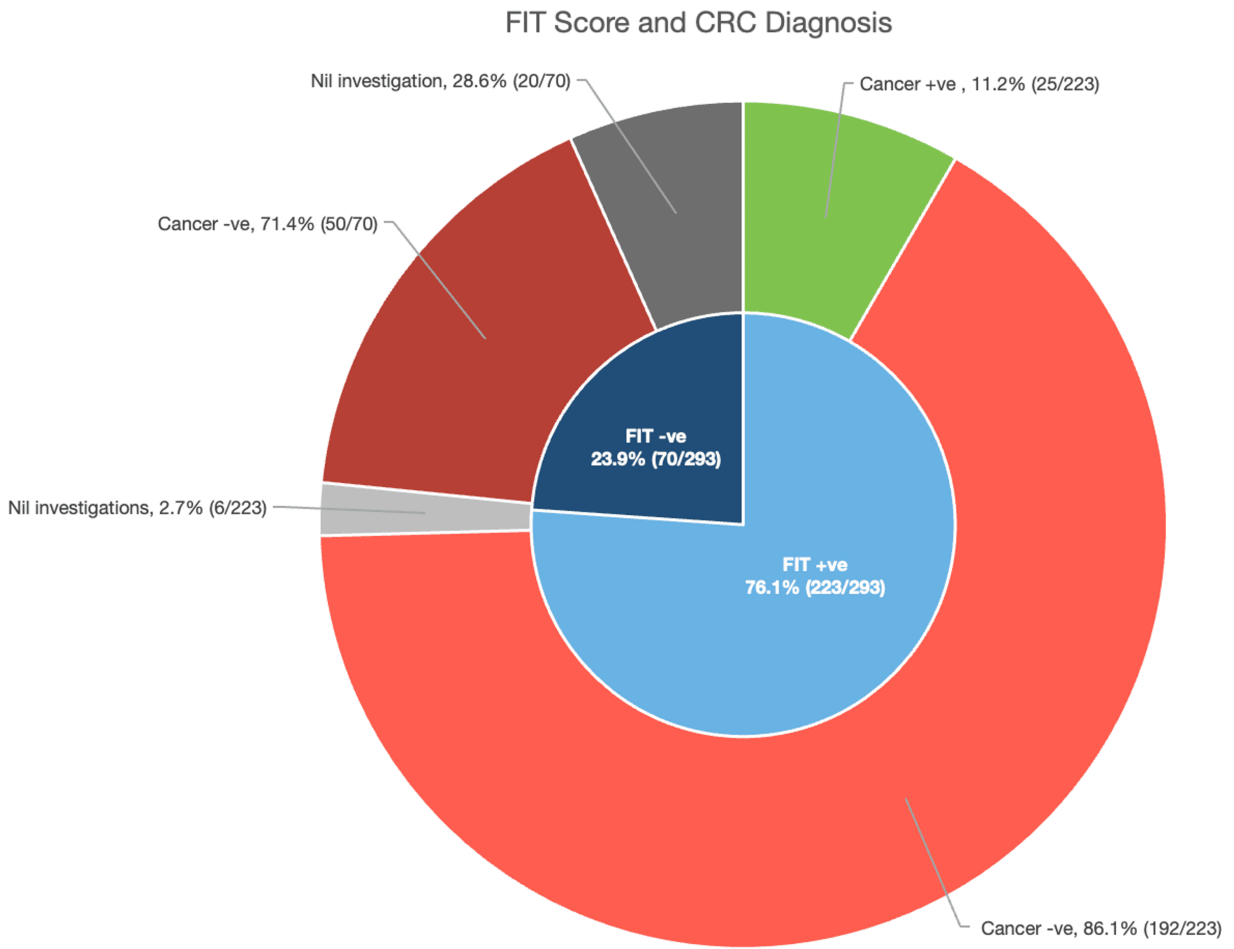
Correlation between FIT score and accurate cancer diagnosis FIT: Faecal Immunochemical Test; CRC: colorectal cancer

Out of the 293 patients included in the study, 223 (76.1%) were FIT‑positive and 70 (23.9%) FIT‑negative, with 20 (28.6%) refusing investigations in the FIT-negative group and six (2.7%) in the FIT-positive group (Figure 1). Among these, 25 (8.5%) patients had CRC: all in the FIT-positive group and none in the FIT-negative group. Thus, in our cohort, the FIT sensitivity for CRC was 100%, and the NPV was 100% (no cancers in FIT-negative). The PPV of a positive FIT for CRC was approximately 11.2% (25 cancers among 223 positives), consistent with other studies.

FIT range vs. cancer detection: predictive value and diagnostic performance

Table 1 summarises diagnostic performance metrics (PPV, NPV, sensitivity, specificity) and statistical significance (p-values) for each FIT range. P-values were shown only for ranges with meaningful sample sizes and statistically interpretable results and therefore excluded for intermediate ranges (FIT 51-400 µg Hb/g). FIT <10 (µg Hb/g) demonstrated an excellent NPV of 100% with no cancers identified in this group. In contrast, FIT 11-50 (µg Hb/g) contained 20% (five out of 25 total) of detected cancers, highlighting that low-positive FIT scores do not entirely exclude malignancy.

**Table 1:** Diagnostic performance metrics (PPV, NPV, sensitivity, specificity) and statistical significance (p-values) for each FIT range PPV: positive predictive value; NPV: negative predictive value; FIT: Faecal Immunochemical Test

FIT range (μg Hb/g)	Total	Cancer +ve	PPV	Sensitivity	Specificity	P-value
<10	70	0	0%	0%	79.8%	0.0065
11-50	128	5	3.9%	20%	30.2%	0.0056
51-100	18	0	0%	20%	23%	-
101-150	13	2	15.4%	28%	18.5%	-
151-200	8	1	12.5%	32%	15.7%	-
201-250	5	0	0%	32%	13.7%	-
251-300	3	1	33.3%	36%	12.9%	-
301-350	1	1	100%	40%	12.9%	-
351-400	4	2	50%	48%	12.1%	-
>400	43	13	30.2%	100%	0%	<0.0001

**Figure 2:**
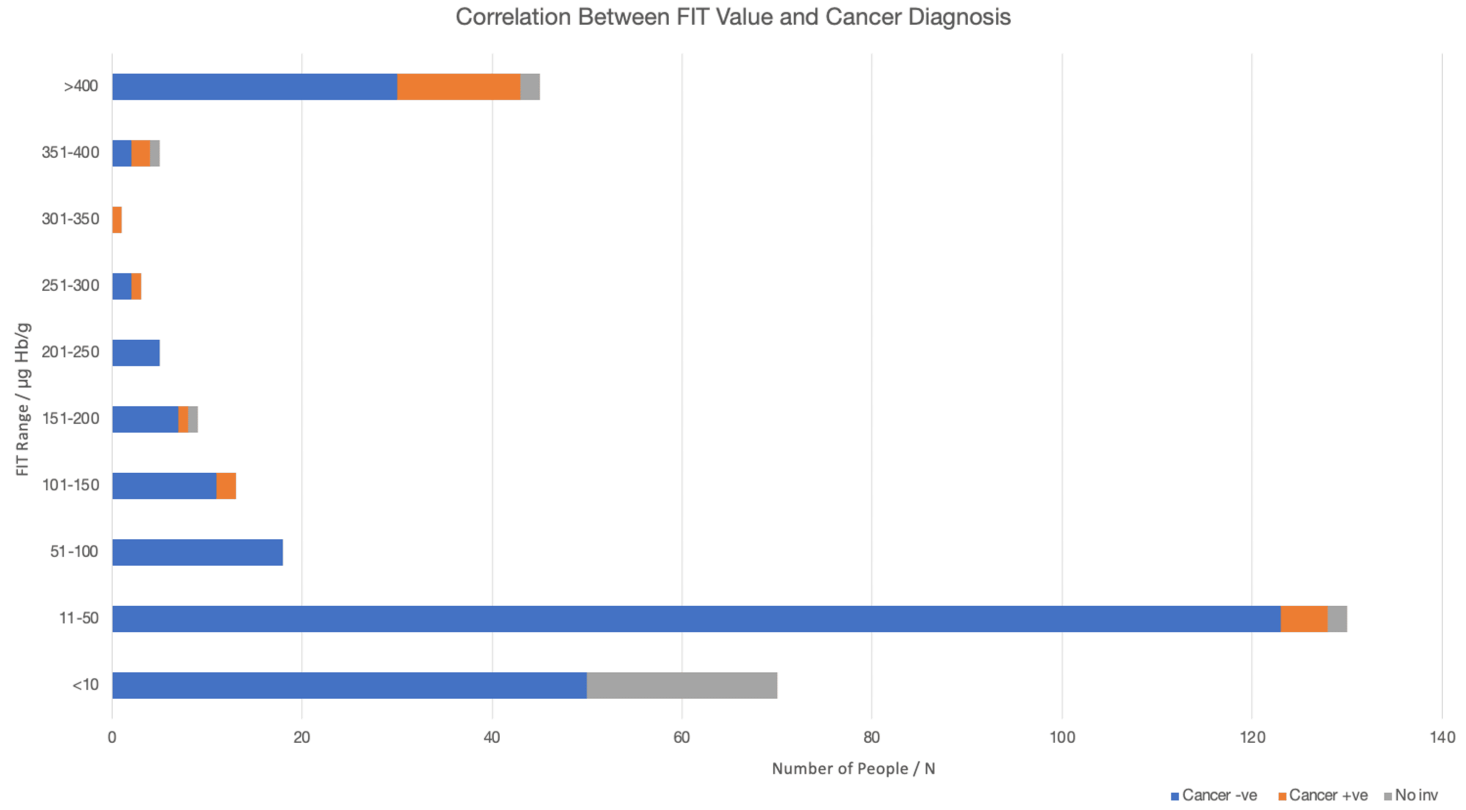
Bar chart showing the distribution of symptomatic patients by FIT score FIT: Faecal Immunochemical Test; No inv: no investigations

The bar chart shows the distribution of symptomatic patients by FIT score, stratified by the presence (orange) or absence (blue) of CRC (Figure 2). The majority of patients had low FIT scores (<50), but cancers were found across a wide range of FIT levels. Most cancers occurred at FIT levels greater than 400 (µg Hb/g), although the PPV remained low overall.

Regional cancer distribution across FIT ranges

**Figure 3:**
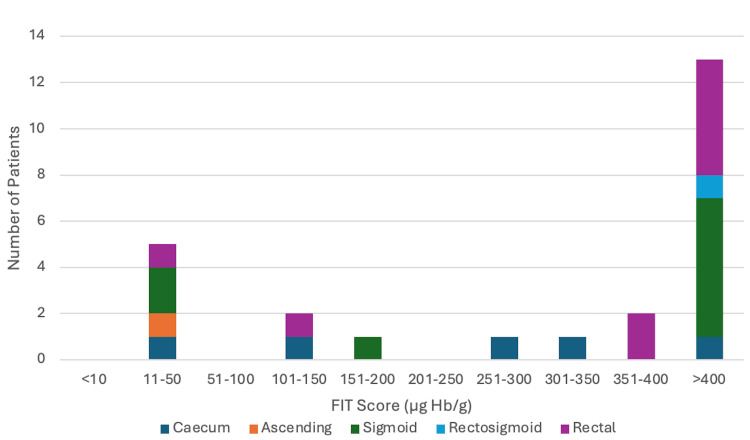
Regional cancer distribution across FIT ranges FIT: Faecal Immunochemical Test

The stacked bar chart shows the anatomical location of CRC stratified by FIT ranges (Figure 3). Left-sided cancers (sigmoid, rectosigmoid, and rectal) accounted for 19 out of 25 (76%) of all CRC in this cohort, with an apparent clustering at higher FIT values. Notably, 12 out of the 19 (63.2%) of left-sided cancers were associated with FIT >400 (µg Hb/g), while only three out of the 19 (15.8%) were found in the lower FIT range of 11-50.

Right-sided cancers (caecum and ascending colon) showed a more dispersed distribution across FIT ranges, including some with low FIT scores, highlighting that a low FIT does not reliably exclude right-sided disease.

Statistical testing of the association between higher FIT scores and left-sided cancer location revealed a non-significant trend (p=0.165), likely reflecting the limited sample size.

Symptom profiles across FIT ranges

**Figure 4:**
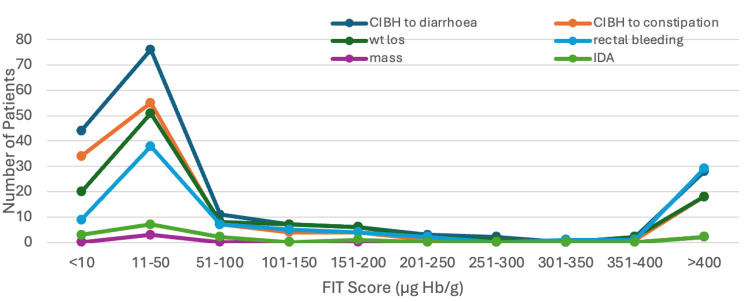
Symptom profiles across FIT ranges FIT: Faecal Immunochemical Test; CIBH: changes in bowel habit; IDA: iron deficiency anaemia; wt los: weight loss

In this cohort of 293 symptomatic patients, change in bowel habit (CIBH) was the most common presenting symptom, reported in 179 (61.1%) patients, and was observed consistently across all FIT ranges without a clear correlation to FIT level (Figure 4). Rectal bleeding in 96 (32.8%) and weight loss in 115 (39.2%) were more prevalent at higher FIT scores, particularly in patients with FIT >400 (μg Hb/g), where 29 out of 96 (30.2%) of rectal bleeding cases occurred. These symptoms were strongly associated with rectal cancers. IDA, although rare with 15 cases out of 293 (5.1%), predominantly occurred in patients with very high FIT scores (>400 μg Hb/g) and was often linked to right-sided cancers. At lower FIT ranges (<50 μg Hb/g), symptoms tended to be more nonspecific, with fewer cases of rectal bleeding or IDA, suggesting that right-sided cancers can present subtly and may be harder to detect in patients with low FIT scores. This pattern highlights the importance of symptom screening in conjunction with FIT.

FIT range as a predictor of benign disease

**Figure 5:**
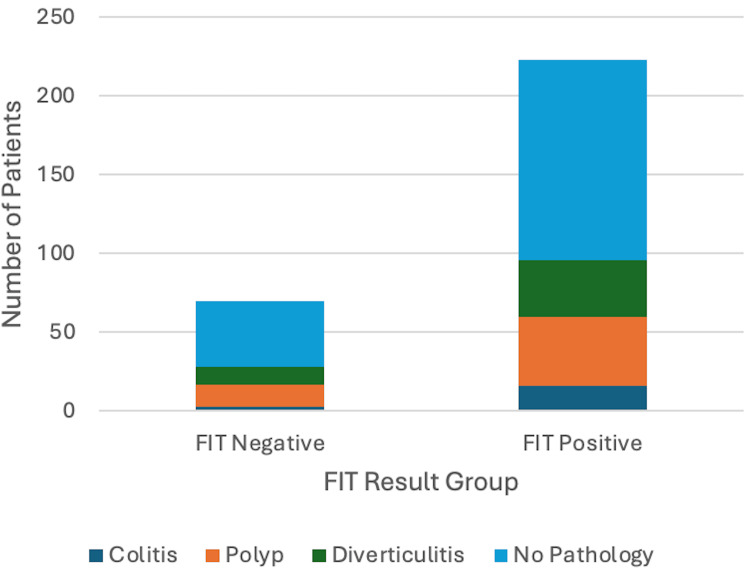
FIT range as a predictor of benign disease: bar chart FIT: Faecal Immunochemical Test

A total of 123 out of the 293 (42%) patients in the cohort had incidental findings of benign bowel pathology, including colitis, polyps, and diverticulitis (Figure 5). These conditions were distributed across both FIT-negative (<10 µg Hb/g) and FIT-positive (>10 µg Hb/g) groups, with polyps and diverticular disease being most prevalent in FIT-positive patients. Colitis, although observed in both groups, was more frequent in FIT-positive patients, with 10 out of 13 cases (76.9%) compared to FIT-negative patients, accounting for only three out of 13 cases (23.1%).

Diagnostic performance analysis revealed that FIT had high sensitivity for benign conditions (colitis: 84.2%; polyp: 75.9%; diverticulitis: 76.6%), but specificity was low across all pathologies (~24%), indicating that a positive FIT result was not specific for benign disease (Table 2). PPVs for benign conditions were low (colitis: 7.2%; polyp: 19.7%; diverticulitis: 16.1%), and, more importantly, none of these benign conditions showed a statistically significant association with a positive FIT result (p>0.05).

**Table 2:** Diagnostic performance of positive FIT score for identifying benign gastrointestinal conditions FIT: Faecal Immunochemical Test

Condition	PPV	NPV	Sensitivity	Specificity	P-value
Colitis	0.072	0.957	0.842	0.245	0.5789
Polyp	0.197	0.8	0.759	0.238	1.0
Diverticulitis	0.161	0.843	0.766	0.24	1.0

These findings confirm that FIT, while designed to detect occult blood in stool, cannot be used as a standalone screening tool for benign bowel pathologies.

FIT range and choice of investigations

**Figure 6:**
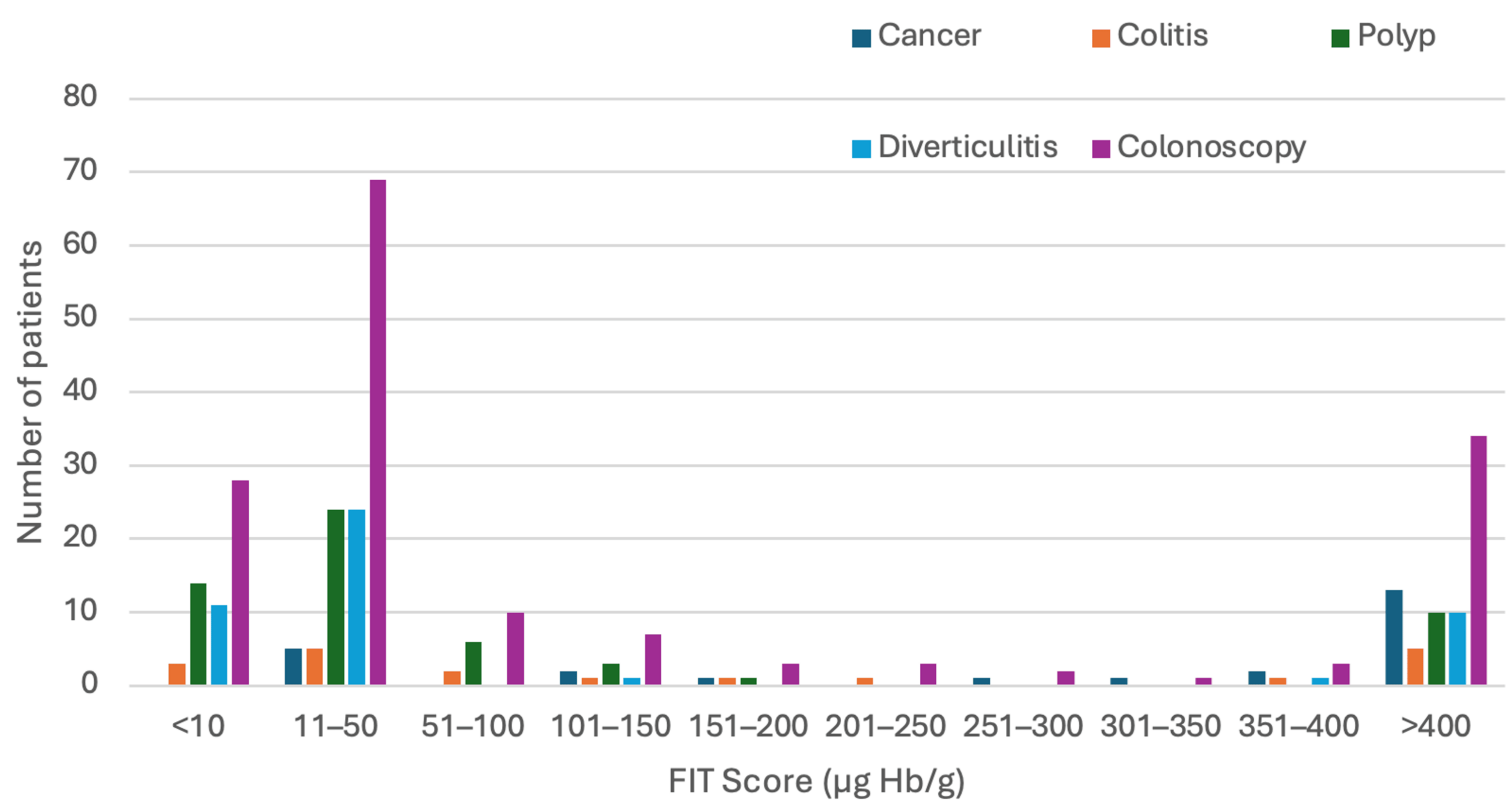
FIT range and choice of investigations: bar chart FIT: Faecal Immunochemical Test

In this cohort of 293 symptomatic patients, diagnostic investigations varied across FIT ranges (Table 3 and Figure 6). Among FIT-negative patients (<10 µg Hb/g; n=70), 28 out of 70 (40%) patients underwent colonoscopy, yet no cancers were detected in this group. In the FIT 11-50 µg Hb/g range (n=128), 69 out of 128 patients (53.9%) had colonoscopies, identifying five cancers, accounting for 20% of all cancers detected in the cohort. While the overall cancer prevalence in this FIT range was low (3.9%), these findings highlight that a low-positive FIT result does not reliably exclude malignancy. Clinical decision-making in this group should not rely on FIT alone; symptom severity and risk factors remain essential for guiding investigation choices.

**Table 3:** Distribution of diagnostic investigations in the cohort CT: computed tomography

Investigation	Total
Colonoscopy	160
CT colonography	66
CT	137
Flexi-sigmoidoscopy	21

In higher FIT ranges, colonoscopy rates remained high; in the FIT 101-150 (µg Hb/g) group, seven out of 13 patients (53.8%) had colonoscopies, detecting two cancers; and in those with FIT >400 (µg Hb/g), 34 out of 43 patients (79.1%) underwent colonoscopy, identifying 13 cancers (30.2%), making this group the most predictive for malignancy.

These findings demonstrate that while colonoscopy was performed frequently across all FIT ranges, the diagnostic yield for cancer was limited in lower FIT ranges (<150 µg Hb/g). This highlights the potential for FIT to guide more selective use of colonoscopy and reduce unnecessary procedures in low-risk patients while ensuring that cancers in low-positive FIT groups are not missed.

## Discussion

CRC is one of the most prevalent cancers globally, ranking as the third most commonly diagnosed cancer and the second leading cause of cancer-related mortality worldwide [4]. The incidence of CRC varies geographically, with higher rates in developed countries attributed to dietary habits, sedentary lifestyles, and ageing populations [5]. In the UK, CRC is the fourth most common cancer, with over 42,000 new cases diagnosed annually [6].

Several risk factors contribute to the development of CRC, including modifiable factors such as high red and processed meat intake, obesity, alcohol consumption, and smoking, as well as non-modifiable factors like age, family history, and genetic predisposition [7]. Conditions such as inflammatory bowel disease, including Crohn's disease and ulcerative colitis, significantly increase the risk. Symptoms often include rectal bleeding, altered bowel habits, abdominal pain, weight loss, and IDA, which frequently occur in the later stages, underscoring the importance of early detection [8].

The early detection of CRC is critical to improving prognosis, and screening programs have become integral in this effort, with the FIT emerging as a valuable tool for population-based screening due to its non-invasive nature and ease of use. FIT has shown higher sensitivity and specificity than traditional faecal occult blood tests (FOBT) and is particularly effective for detecting left-sided CRC and advanced adenomas [9].

However, FIT's diagnostic accuracy depends on symptomatology and thresholds for haemoglobin concentration in stool samples [10]. Colonoscopy remains the gold standard for definitive diagnosis, providing the opportunity for both the detection and removal of precancerous lesions during the same procedure [11]. Other modalities, such as CTC and flexible sigmoidoscopy, are valuable adjuncts, particularly for patients unable to tolerate colonoscopy [12].

Management for CRC depends on the stage at diagnosis. Early-stage cancers confined to the bowel wall may be treated effectively with surgical resection alone, while more advanced stages typically require adjuvant chemotherapy to reduce the risk of recurrence [13].

FIT has become a cornerstone in the screening and early detection of CRC, particularly due to its non-invasive nature and ease of use. FIT is especially effective in identifying left-sided CRC, as confirmed by previous studies [14]. Screening programs utilising FIT have shown considerable promise in reducing CRC incidence and mortality by facilitating earlier diagnosis and intervention [15]. However, FIT sensitivity can vary depending on the location and stage of the cancer, necessitating the integration of additional diagnostic modalities to improve detection rates.

Earlier studies have already proven that FIT screening is both a sensitive and specific test for the detection of CRC, as seen in a study conducted by Digby et al. [16], which showed a graded relation between faecal haemoglobin concentrations and CRC detection rates. In addition, Goede et al. [17] have reaffirmed that incorporating FIT scores into clinical algorithms can prioritise patients for colonoscopy, thereby streamlining the effectiveness and cost-efficiency of screening programs. This remark underscores the expanding role of FITs in individualised screening strategies, especially when coupled with other modalities to augment early detection and decrease mortality from CRC.

This retrospective cohort study reinforces the value of FIT as an effective rule-out test in symptomatic patients, confirming its high NPV for CRC among FIT-negative individuals (<10 µg Hb/g). However, the findings also highlight that FIT thresholds should be interpreted with caution, since a substantial proportion of cancers were detected among patients with low-positive FIT results (11-50 µg Hb/g), including right-sided malignancies. These findings underscore the importance of integrating FIT results with clinical judgement and symptomatology to guide decision-making. Careful consideration of symptoms can help identify patients at risk of cancer, including those with low FIT scores, while also avoiding unnecessary invasive investigations such as colonoscopy in low-risk individuals.

As a retrospective, single-centre cohort study with a relatively small sample size (n=293), we acknowledge limitations in the generalisability of our findings. The study relied on real-world clinical data, and symptom reporting may have been inconsistent or incomplete. Not all patients underwent full investigation, particularly those with low FIT scores or who declined procedures, introducing potential verification bias. Additionally, the uneven distribution of cancer cases across FIT ranges reduced statistical power to detect significance in some subgroups. Being a retrospective study, we were unable to provide detailed methodology regarding FIT specimen handling, laboratory processing, or quality control, as all tests were performed in primary care prior to referral. Potential confounders, including age, comorbidities, and medication use, were not adjusted for, which may affect the observed associations. Future research would benefit from larger, multi-centre cohorts and strategies to optimise the combined use of FIT with adjunctive diagnostic tools to enhance diagnostic accuracy and reproducibility.

## Conclusions

FIT is a widely used triage tool for symptomatic patients suspected of CRC. This study aimed to assess whether lower FIT values correlate with a reduced risk of cancer to support prioritisation of investigations. Ultimately, the findings from this study reinforce FIT's role as a reliable rule-out test for CRC while also highlighting that low-positive FIT results do not guarantee safety. While most cancers were identified in patients with FIT >400 (µg Hb/g), the PPV remained modest overall, and high FIT scores did not always indicate malignancy. Over 160 colonoscopies were performed across all FIT groups, including in the FIT-negative group, yet most did not result in cancer diagnoses. These findings demonstrate that FIT alone cannot reliably predict cancer or benign pathology and should be interpreted alongside symptom assessment to guide investigation choices and avoid unnecessary invasive procedures. This supports a complementary approach, where FIT serves as an initial triage tool to prioritise colonoscopy referrals and optimise healthcare resource allocation. However, these findings are limited to a single-centre retrospective cohort and should be interpreted in that context.
